# Effective Dispersal of Caribbean Reef Fish is Smaller than Current Spacing Among Marine Protected Areas

**DOI:** 10.1038/s41598-017-04849-5

**Published:** 2017-07-05

**Authors:** Diana M. Beltrán, Nikolaos V. Schizas, Richard S. Appeldoorn, Carlos Prada

**Affiliations:** 1 Department of Marine Sciences, University of Puerto Rico, Mayagüez, Call Box 9000, Mayagüez, 00681 Puerto Rico, USA; 2 Smithsonian Tropical Research Institute, Box 2072, Balboa, Panama

## Abstract

The oceans are deteriorating at a fast pace. Conservation measures, such as Marine Protected Areas, are being implemented to relieve areas from local stressors and allow populations to restore to natural levels. Successful networks of MPAs operate if the space among MPAs is smaller than the dispersal capacity of the species under protection. We studied connectivity patterns across populations in a series of MPAs in the common yellowhead Jawfish, *Opistognathus aurifrons*. Using the power of genome-wide variation, we estimated that the maximum effective dispersal is 8.3 km. We found that MPAs exchange migrants likely via intermediate unprotected habitats through stepping stone dispersal. At scales >50 km such connectivity is decreased, particularly across the Mona Passage. The MPA network studied would be unable to maintain connectivity of these small benthic fishes if habitat in between them is extirpated. Our study highlights the power of SNPs to derive effective dispersal distance and the ability of SNPs to make inferences from single individuals. Given that overall reef fish diversity is driven by species with life histories similar to that of the yellowhead jawfish, managers face a challenge to develop strategies that allow connectivity and avoid isolation of populations and their possible extinction.

## Introduction

Tropical ecosystems have degraded in the last decades as a result of human activities. This decline is marked on coral reefs, where species commonly seen in the 1970s are rarely observed today, especially apex predators^1^. One conservation initiative to restore marine populations is the designation of networks of Marine Protected Areas (MPAs), which in their most restrictive form are no-take. No–take MPAs are fishing-free spaces that, when properly managed can restore populations if no other stressors are present. These MPAs act in multiple ways to increase the spawning potential of overexploited species, but they also have associated direct benefits to ecosystem as a whole^2^. Thus, while MPAs were originally focused on the restoration of fished species, they are now used to protect biodiversity on reefs^3^. Networks of MPAs are conceptually based on the idea that most marine populations are ecologically connected by the dispersal of planktonic larvae^4^. The spatial scale of larval dispersal determine the degree of connectivity among marine populations, providing information on the ideal reserve size and the minimum spacing among reserves to achieve both self-recruitment and connectivity^5^. Determining the scale of this effective movement of larvae is critical to designing successful MPA networks.

In theory, most marine systems lack obvious barriers to dispersal and MPAs should be connected and work as networks over large spatial scales^4^. Reproduction in marine systems usually results in planktonic larvae that disperse from days to months in the ocean, potentially connecting populations over hundreds of kilometers. Early biophysical models of larval dispersal in the surface ocean have often found marine connectivity across populations^4, 6^, and genetic inferences have provided evidence of gene flow over thousands of kilometers^7, 8^. However, recent studies have challenged previous assumptions of high connectivity among marine populations over large spatial scales^9–13^.

Recent physical oceanographic models suggest that populations are locally maintained and connectivity among populations restricted^14–16^. Understanding whether populations are connected and the spatial scale at which this occurs is central to designing effective MPA networks. Estimating larval dispersal is challenging, as common mark-recapture studies do not work efficiently in populations that produce millions of planktonic larvae that quickly dilute in the sea and almost all of which die. Fortunately, genetic surveys provide a powerful indirect estimate of population connectivity^13^. If populations are connected, genetic variation across populations should be minor, but if genetic exchange is restricted and migrants are unable to reproduce in the new population^17^, genetic differentiation among populations develops^18^. Now, easy to gather genomic data can capture subtle spatial genetic variation, revealing the effective movement of migrants and their per generation dispersal^19^.

Connectivity inferred from genetic data often reflects the product of evolutionary processes across multiple time scales in the history of a species, including those at ecological scales, which are the most useful for designing MPA networks. Fortunately, we can measure connectivity at different time scales by applying different techniques. Gene genealogies within a coalescent framework integrate migration rates over time (e. g. whole history of a population) and provide long-term estimates of migration^20^. Conversely, inferences based on linkage disequilibrium^21^ can capture more contemporary connectivity.

We used genome-wide Single Nucleotide Polymorphisms (SNPs) and microsatellites to test whether existing MPAs in the northeast Caribbean are genetically connected through migration among them, and large enough to sustain marine populations of the yellowhead Jawfish (*Opistognathus aurifrons*). In theory, populations of *O*. *aurifrons* should readily connect thanks to its three-week pelagic larval duration (about 400–500 km dispersal capacity)^15, 22, 23^. We studied genetic variation across a longitudinal gradient of seven MPAs that span the Mona Passage, a well-known biogeographic barrier between Puerto Rico and Dominican Republic^9, 24^. We first inferred genetic diversity and estimated effective population size in the common reef fish, *O. aurifrons*. We then quantified variation across geography and estimated the per generation effective dispersal distance using *in-situ* fish densities. Finally, we tested for differences between contemporary and historical connectivity across these MPAs.

Our data suggest an average effective movement of 10 km per generation. MPAs are connected at scales smaller than 100 km, likely as a result of migrants moving across generations through intermediate unprotected reef areas. For species with life histories similar to that of the yellowhead jawfish, our data indicate that current MPAs are too far apart to maintain connectivity over short time frames, which puts at risk the long-term conservation of the diverse, small benthic fish assemblage as represented by the yellowhead jawfish.

## Results

### Genetic Diversity

To measure connectivity, we sampled populations in a network of MPAs along the southern (Caribbean) side of Puerto Rico and the Dominican Republic (Fig. S1). On average, the geographic distance between any two sampled MPAs is 90 km, with the largest being 486 km. A total of 260 individuals were collected from seven MPAs separated by the Mona Passage - two in the Dominican Republic: Parque Submarino La Caleta, and Parque Nacional Natural del Este, and five in Puerto Rico: Mona Island Natural Reserve, Desecheo Island Marine Reserve, Tres Palmas Marine Reserve, La Parguera Natural Reserve, and Canal Luis Peña Marine Reserve at Culebra Island. We genotyped all individuals at 12 newly designed microsatellites^25^ (Tables S1 and S2) and a subset of 54 individuals from all seven populations at 10,047 SNPs (Tables S3 and S4).

### Genetic variation segregates gradually across geography

**Figure 1:**
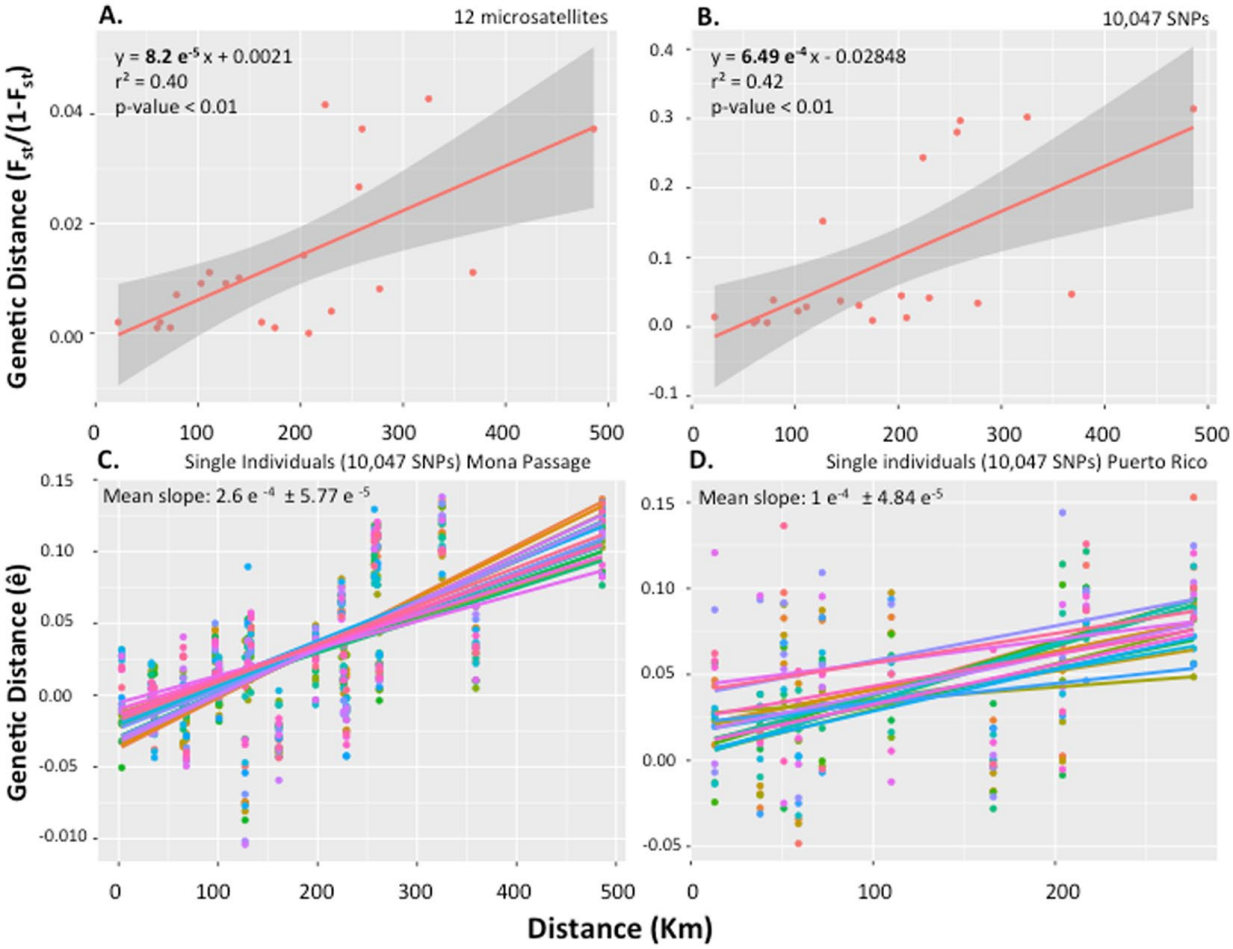
Isolation by Distance (IBD) in *Opistognathus aurifrons* from seven sampling sites along Puerto Rico MPAs (La Caleta, PNNE, Mona Island, Desecheo Island, Tres Palmas MR, La Parguera and Culebra). (**A**) IDB from 12 microsatellites; (**B**) IBD from 10,047 SNPs and (**C**) IBD from single individuals for seven sampling sites (La Caleta, PNNE, Mona Island, Desecheo Island, Tres Palmas MR, La Parguera and Culebra). (**D**) IBD from single individuals for five MPAs within Puerto Rico (Mona Island, Desecheo Island, Tres Palmas MR, La Parguera and Canal Luís Peña at Culebra) with 10,047 SNPs.

Genetic variation among populations across the MPA network is partitioned by geography (Fig. 1 and Table 1). Geography explains 40% (p < 0.05) of the variation for microsatellites and 42% (p < 0.05) for SNPs. Estimates from single individuals are also remarkably similar (40%; p < 0.05) and support the use of SNPs to sample fewer individuals per population, ameliorating the effects on wild fauna.

**Table 1:** Spatial genetic structure (*F*_*ST*_) across the Mona Passage as derived from 12 microsatellites (above diagonal) and 10,047 SNPs (below diagonal).

	Tres Palmas	La Parguera	PNNE	Mona Island	La Caleta	Desecheo	Culebra
Tres Palmas	—	0.002	0.002	0.001	**0.026**	0.002	0.005
Parguera	0.009	—	**0.014**	**0.011**	**0.041**	**0.007**	0.001
PNNE	**0.03**	**0.043**	—	**0.009**	**0.009**	**0.010**	**0.011**
Mona Island	0.006	**0.028**	0.022	—	**0.040**	0.001	**0.008**
La Caleta	**0.219**	**0.232**	**0.132**	**0.196**	—	**0.036**	**0.036**
Desecheo	0.014	**0.037**	0.036	**0.006**	**0.229**	—	0.004
Culebra	**0.013**	**0.009**	**0.045**	**0.033**	**0.239**	**0.04**	—

Alpha values were adjusted with a Bonferroni correction (0.05/21 = 0.023). Significantly different pairwise comparisons are shown in bold (p < 0.01).

As a result of the geographic isolation, pairwise *F*_*ST*_s were highest when comparing populations from West (La Caleta and PNNE, both in the Dominican Republic) and East of the Mona Passage (p < 0.01). The Mona population also showed genetic differentiation when compared to all other populations (p < 0.01), except when compared with Tres Palmas (Table 1).

At finer scales, *F*_*ST*_s from SNPs suggest population differentiation at all levels (except Desecheo vs. Tres Palmas and Mona vs. Tres Palmas). The change in allele frequencies detected by pairwise *F*_*ST*_s suggests high geographic structure even at scales smaller than 100 km. Our comparison of *F*_*ST*_s between microsatellites and SNPs suggests that SNP data provide higher resolution (Table 1). For instance, significance of pairwise *F*_*ST*_s from microsatellites disappeared in six of the pairwise population comparisons, while it was captured by SNPs.

**Figure 2:**
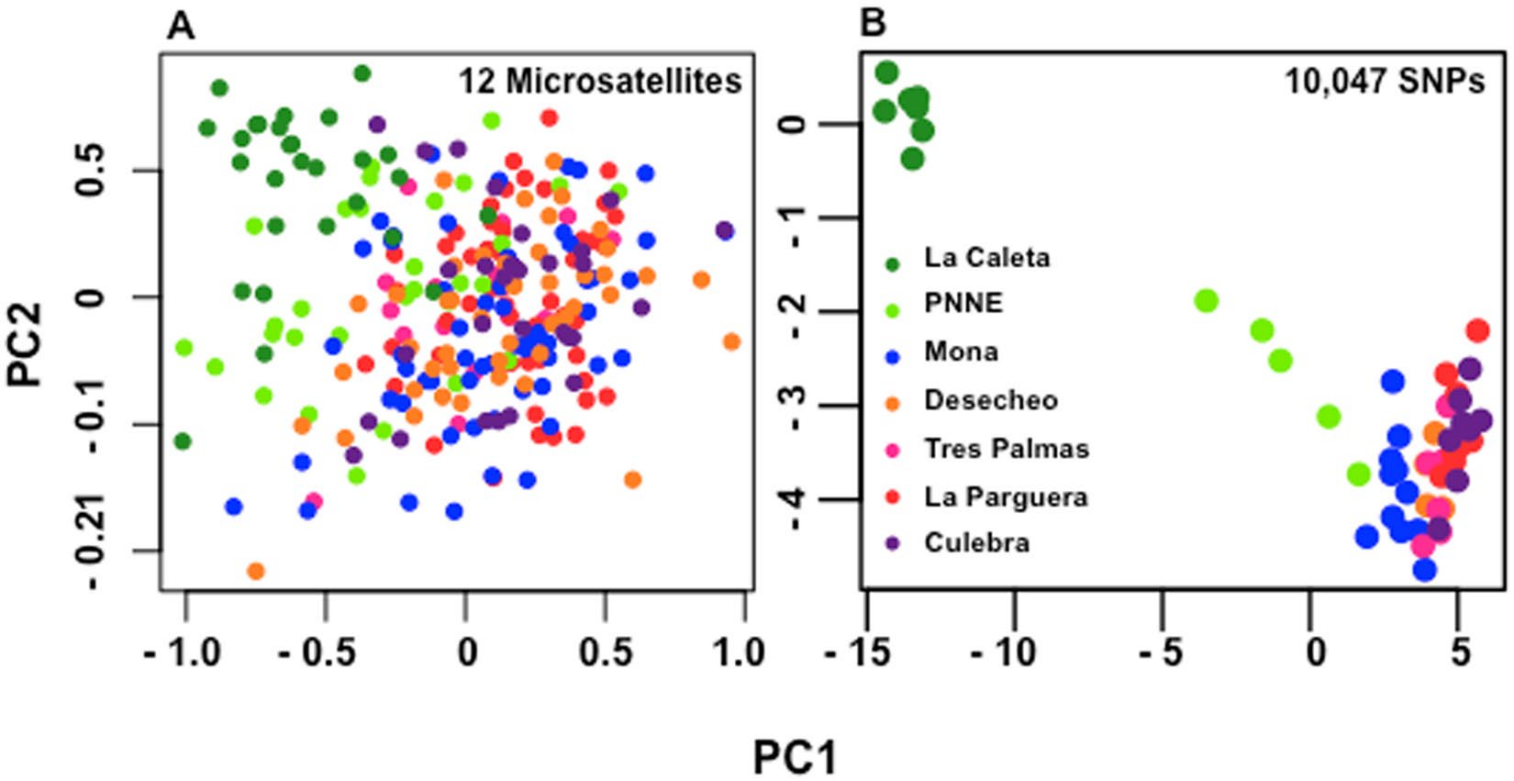
Multidimensional summary of genetic variation. (**A**) Principal component analysis (PCA) of the individuals genotypes with 12 microsatellites. (**B**) Principal component analysis (PCA) of the individuals genotypes with 10,047 SNPs.

Principal component analysis also shows the geographical partition of genetic variation and the subtle but noticeable genetic break within the Dominican Republic (Fig. 2). It also depicts the overlap in genetic variation at Mona Island from individuals sampled in the Dominican Republic and those sampled in western Puerto Rico. The first two principal components explain 72% and 18% of the genetic variation. Component one separates populations from west to east, while component two segregates populations within Puerto Rico (Fig. 2A). Results from both microsatellites and SNPs are similar. However, the SNP-derived PCA clearly delineates at least two tight groups: La Caleta of Dominican Republic and Puerto Rico (Fig. 2B). The PNNE population, while geographically close to Puerto Rico (Fig. 2B) is quite spread, with high intra-sample variability. The >10 k SNPs have by far the highest resolution, with reduced within-group variation and increased among-group distance.

### Effective dispersal is less than ten kilometers

Density of fish from field surveys ranged from 5,554–74,400 fish per km^2^ ^26, 27^. As expected these estimates are biased towards high densities as the surveys were conducted in habitats in which the species is common. In contrast, our estimates from genetic data are 21–282 times lower. The effective population size from 12 microsatellite loci as estimated by IMa2^28^ varied from 1,000,290 to 34,318,231 (HPD in IMa2) with a mean of 5,119,320 breeding individuals across the whole Caribbean (26,000 km^2^), with a density of 263.4 fish per km^2^. We considered a microsatellite mutation rate of 5 × 10^−4^ per locus per year a value commonly used in the literature^29–31^ and one year per generation. Applying these density estimates to Rousset’s equation, the effective dispersal distance of the yellowhead jawfish ranges from 0.1 to 3.4 km. To avoid the biased increase in slope due to the genetic break between the Dominican Republic and Puerto Rico, we also inferred isolation-by-distance (IBD) patterns only within Puerto Rico (Fig. 1D). If we use the smaller IBD slope obtained from samples only within Puerto Rico and the lowest density, the effective dispersal distance is 8.3 km.

### Restricted contemporary gene flow contrasts with historical migration across the Mona Passage

**Figure 3:**
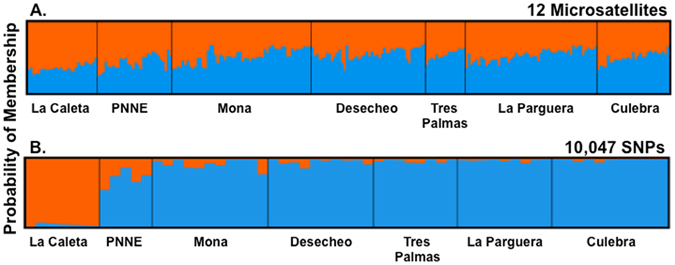
Graphical summary of clustering Analysis for *Opistognathus aurifrons* genotypes from seven Marine Protected Areas along Mona Passage using Structure. Probability of Membership of each sampling site (n = 7) from individuals per site scored with 12 microsatellites (**A**) and 10,047 SNPs (**B**). Each vertical line represents an individual and the estimated proportion of the individual’s genome from each inferred cluster.

To further describe the genetically isolated groups, we used linkage disequilibrium across markers. STRUCTURE and Admixture analysis suggests the presence of two genetic pools across the sampled area, represented as orange and blue in Fig. 3 (Fig. S5). The power to assign individuals to two groups differs between microsatellites and SNPs. SNP analysis clearly delineates the two pools. One gene pool is confined to populations in Puerto Rico, including Mona Island, while the other consist of La Caleta, Dominican Republic. The PNNE is an admixed population from La Caleta and Puerto Rico. The pattern is subtler for microsatellites. The orange genotype is more common in the Dominican Republic and is replaced gradually by the blue genotype as one moves eastward to Puerto Rico (Fig. 3). Estimates of the proportion of individuals displaying the orange genotype is significantly different when comparing populations from the Dominican Republic and those from Puerto Rico (p < 0.001). Self-recruitment estimates (>0.6) from BayesAss using SNP data also supports limited connectivity among populations. The only significant genetic exchange occurs among populations within Puerto Rico. However, the lack of consistency across datasets does not allow to precisely estimating the magnitude and the geographic center of such migration (Fig. S3 and Table S5).

**Figure 4:**
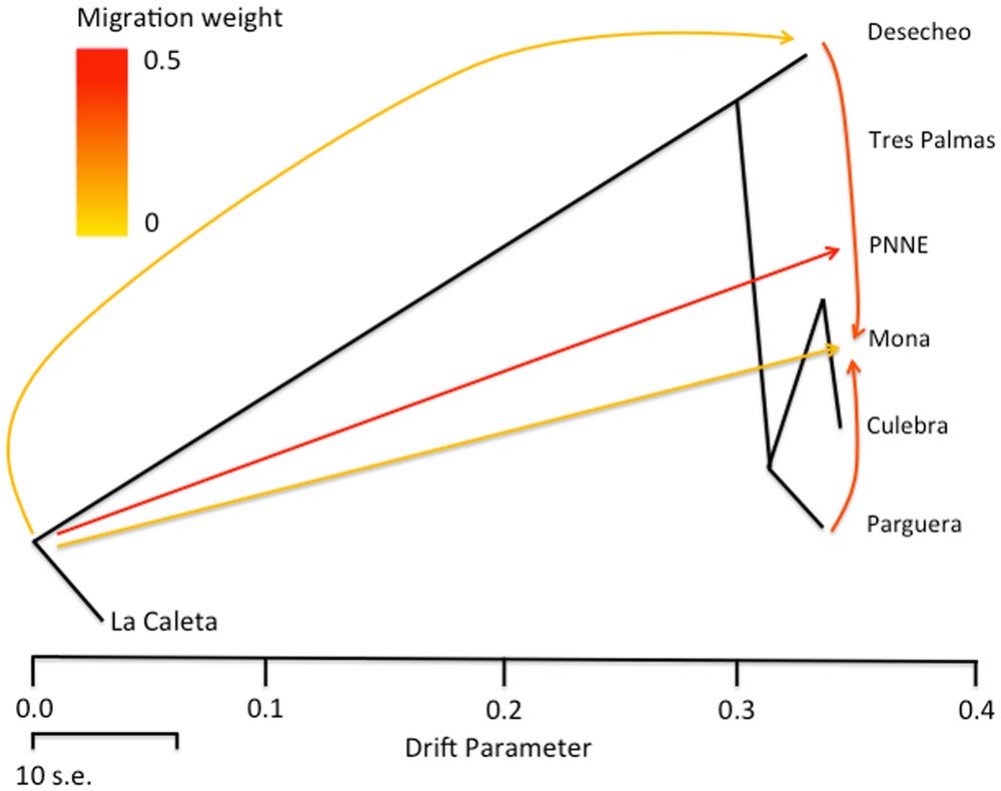
Phylogram of the inferred *Opistognathus aurifrons* population relationships. Scale bar shows 10 times the average standard error of the estimated entries in the sample covariance matrix. Arrows depict migration patterns. Red arrows represent stronger migration.

Sharp contemporary genetic structure contrasts with historical gene flow estimates across the Mona Passage (Fig. 4). We found extensive migration across the Mona Passage. Historical migration seemed higher in the eastern direction from the Dominican Republic to Mona and Puerto Rico. There is also a strong historical gene flow signal from elsewhere in Puerto Rico to Mona, making Mona a historical stepping-stone connecting Puerto Rico and the Dominican Republic.

## Discussion

The benefits of creating networks of MPAs are evident when the spacing among protected areas is small enough to allow larvae of neighboring populations to achieve genetic connectivity^5^. It is thus critical to estimate the magnitude and direction of genetic exchange and evaluate their contemporary versus historical component. To understand contemporary and historical connectivity among populations, we studied genetic variation across a Caribbean network of MPAs in a common benthic reef fish with pelagic larvae. Analyzing variation at 12 microsatellite loci and over 10k SNPs, we showed that: (i) The yellowhead jawfish has a large amount of genetic variation with an effective population size on the order of millions of individuals. (ii) Effective migration distance per generation is less than 8.3 kilometers generating a pattern of genetic segregation by geography. (iii) Contemporary gene flow is restricted across the Mona Passage, segregating areas into two populations: those west of Mona, and all others. (iv) Contemporary genetic structure across the Mona Passage contrasts the historically high gene flow in both eastern and western directions.

In the broadest sense our genetic analyses suggest populations within the studied MPA network are connected in a stepping-stone fashion via intermediate unprotected reef habitats. Effective dispersal within Puerto Rico of 10 km allows only neighboring populations to exchange migrants, and such limited connectivity results in a positive correlation between geographical and genetic distances as predicted from theory^32^. Across the Mona Passage, the effective dispersal distance was substantially lower: 4 km. Below we present a perspective on the current spacing and connectivity among these already designated MPAs, the critical role of the Mona Island Reserve to preserve connectivity across the Passage and the contrasting patterns of historical and contemporary connectivity across this network of MPAs.

### Current MPAs are too far apart to allow connectivity in the absence of intermediate habitats

Our data suggest a pattern of genetic isolation with geography, with some populations separated only by <100 km showing significant genetic differentiation. Our effective dispersal distance estimates also suggest the fish would move less than 10 km per generation, providing an upper bound for the largest spacing among, MPA networks if constant and full connectivity are necessary design criteria. Our dispersal estimates fall within previous estimates of fish dispersal such as for *Stegastes partitus* (9 km), *Hypoplectrus nigricans* (10 km) and *Hypoplectrus puella* (2–14 km)^33, 34^ and the anemone fish *Amphiprion clarkii* (4–20 km)^35^, but lower than in *Thalassoma bifasciatum* (27 km), *Haemulon flavolinueatum* (46 km) *Chaetodon capistratus* (52 km)^34^.

Dispersal estimates contrast with the mean distance within the network of MPAs sampled (90 km) and all Management Areas (37 km) in the studied area, regardless if they are enforced or not. This is 8X (or 3X if we include all Management Areas) the ideal space among MPAs to ensure connectivity and a high level of protection. Our study suggests that the sustainability of MPAs separated by >20 km may exist only because of the existence of intermediate unprotected habitats. Continued decline in reef health, however, may result in the disappearance of those unprotected habitats, resulting in fragmented isolated populations prone to demographic stochasticity and extinctions.

Our results also highlight the power of SNP data to derive estimates from single individuals. Slopes calculated from population level data and single individuals were almost identical. Finding similar genetic patterns from fewer individuals diminishes harm to wild fauna in genetic surveys and thus preferable for conservation biology.

### Mona Island as a link between the east and the west of the Caribbean

We found a subtle genetic break west from the Mona Passage, dividing populations within the Dominican Republic, with Mona Island being part of the eastern cluster with unequivocal western genetic traces in some individuals. Historically, however, gene flow has occurred between the two populations, and Mona Island is a critical link that has allowed long-term migration in both east and west directions (Fig. 4). Our results fit the east-west pattern of differentiation, probably generated by the effect of deep ocean water in creating undesirable conditions, which limit larval migration across the Passage. For instance, in summer currents flow southward along the Dominican Republic and northward around Puerto Rico, while in winter surface water (up to 50 m) flows northward and deeper water (below 50 m) flows southward^14, 16^. Such seasonal patterns of the currents are believed to limit larval dispersal across this narrow stretch during certain times of the year^14^. The population differentiation in the yellowhead jawfish highlights the importance of Mona Passage as shown in genetic studies of *Acropora* corals^14, 24, 36^ in a damselfish and a wrasse^37^, goby fishes^9, 38^, and also with morphometric and color pattern variations in populations of fishes^39, 40^. Our detailed sampling, however, revealed the biogeographic boundary in *O*. *aurifrons* occurs between La Caleta and PNNE within the Dominican Republic.

### Limited gene flow at ecological scales despite historical long-term connectivity

Our historical estimates of genetic variation in the yellowhead jawfish suggest that the current genetic break in the Dominican Republic is a recent barrier and connectivity could be restored as has happened in the past. Variation between contemporary genetic structure and historical gene flow has also been found in the whitesnout anemonefish *Amphiprion mccullochi* in southeastern Australia^41^. One caveat from this study is the use of the program Migrate-n^42^ on microsatellite data for contemporary gene flow. Migrate-n uses the harmonic mean to integrate variation in gene flow over evolutionary times scales and provides an estimate of the long-term migration rate^43^. In contrast, we calculated long-term migration using Treemix 1.12^44^ as a measure of historical gene flow and STRUCTURE 2.3.4 and Admixture 1.3^45, 46^ to estimate recent genetic structure.

The genetic barrier around the Dominican Republic and Mona is a temporary barrier where over longer time scales the major westward current does not impede the flux of migrants going eastward. The high gene flow across evolutionary scales is not surprising because the yellowhead jawfish has a pelagic larval duration of more than two weeks^22^, which could potentially allow the fish larvae to travel 400–500 km in a single generation^15^. While contemporary gene flow is of most use for conservation, our results stress the role of historical migration in shaping contemporary patterns. In the context of MPA theory, this suggests that present-day barriers to gene flow may hide the historical potential of dispersion of the species. Critically for MPA design, the PNNE and Mona Island represent mosaics of the genetic variation from the east and the west and have historically function as stepping-stones to connect those populations. Preserving Mona is a large step forward towards keeping a condensed amount of the Caribbean genetic diversity and the likelihood of connectivity between the east and the west, if oceanographic conditions allow and conditions in the existing MPAs do not degrade further.

Our results suggest MPAs along the Mona Passage only exchange migrants only through intermediate populations in unprotected habitats. Our findings stress the importance of creating intermediate MPAs to generate a network that ensures connectivity. Our contrasting pattern of contemporary genetic differentiation and historical gene flow highlights the importance of preserving populations, such as the Mona population, at the edges of current genetic breaks. Mona Island is a reservoir of genetic variation from both the eastern and western population and a critical link to allow pulses of gene flow over longer time scale.

## Conclusions

**Figure 5:**
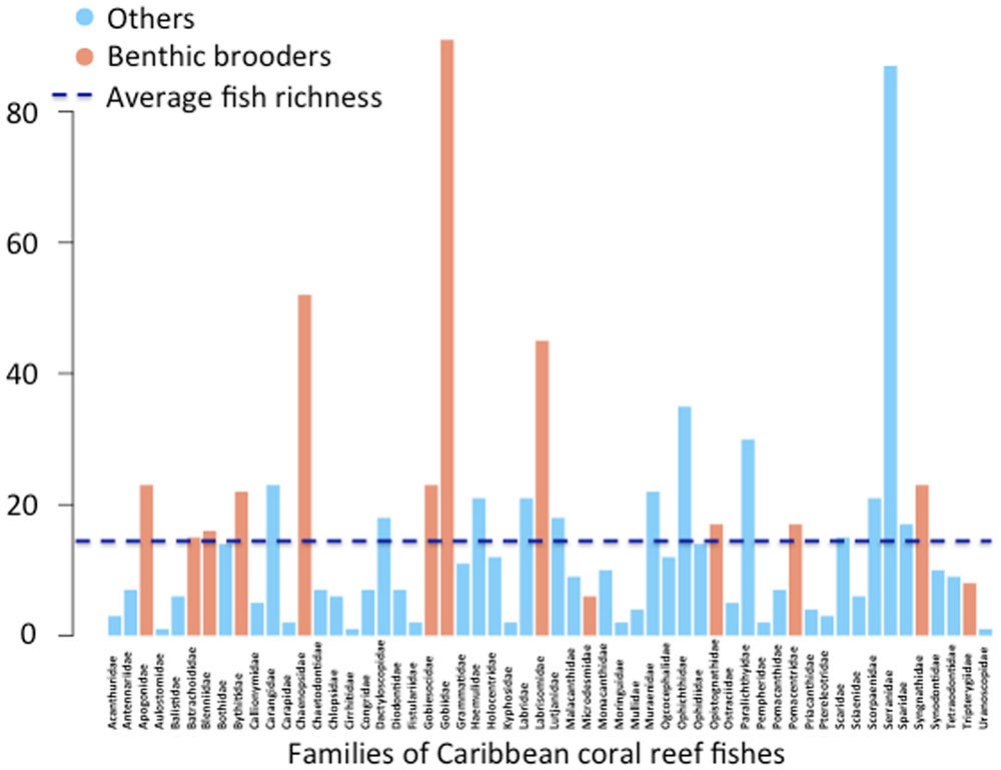
Number of species per Caribbean reef fish families.

Species diversity is driven by small benthic taxa such as the yellowhead jawfish (Fig. 5). Small benthic fish dominant on coral reef systems have an effective dispersal distance less than ten kilometers. MPAs are now widely used to preserve fish diversity across the globe, particularly across coral reef habitats. To use MPAs to protect fish biodiversity, and maintain the health of coral reefs, MPAs need to be closer.

## Materials and Methods

### Studied Species

To estimate effective dispersal among MPAs, we chose the benthic yellowhead jawfish (*Opistognathus aurifrons*), a common Caribbean species^47^. The yellowhead jawfish is a sedentary benthic fish that lives in decorated burrows on calcareous sand at depths between 3–50 m. Spawning occurs in the burrow and the male orally incubates the eggs^47, 48^. Density estimates for the species are known and available in Puerto Rico^26^ and Panama^27^. Given our goal of capturing subtle genetic changes in a fish with pelagic larvae across a densely sampled geographical area, the benthic jawfish is ideal. Small egg-brooding fishes such as the yellowhead jawfish represent a large portion of the fish biodiversity on coral reefs. MPAs while originally developed to restore fished species, are now used to protect biodiversity on reefs^3^. All experimental procedures followed the regulations of the government of Puerto Rico. Our experimental procedure was approved by the University of Puerto Rico Animal Care and Use Committee, the Puerto Rico Department of Natural and Environmental Resources (permit number: 2011-IC-007) and *Secretaría de Estado y Medio Ambiente y Recursos Naturales* at Dominican Republic (permit number: 0611). In all instances we followed their guidelines and recommendations.

### Molecular Markers and Genotyping

To generate genotypic data, we extracted genomic DNA across all 260 individuals using the QIAGEN DNeasy Kit following the manufacturer’s protocol. We genotyped all individuals at 12 newly designed microsatellites^49^ (Table S1) and a subset of 54 individuals at 10,047 SNPs. We included representatives from all seven populations. Both microsatellite and SNP datasets are available through at Dryad (doi:10.5061/dryad.8gg2p). All specimens were vouchered at the Marine Sciences Fish Collection of the University of Puerto Rico and are available upon request.

To genotype individuals at microsatellite loci, we used our described two-step protocol^49^. All PCR amplicons were visualized using an automated sequencer (ABI 3130xl). An internal size standard (ROX-400) was used to estimate microsatellite sizes. We analyzed chromatograms with size standards and scored fragments in Geneious R8 8.1.4^50^. For spurious scoring peaks, we repeated the PCRs and ran them individually on the sequencer as single samples. Individuals were repeated at least five times before deeming them as failed.

To evaluate deviations from Hardy–Weinberg equilibrium in microsatellite data, we used MICROCHECKER 2.2.3^51^. All loci were under Hardy-Weinberg equilibrium, except loci 1588 and 7983. Genetic differentiation and gene flow were estimated including and excluding these two loci, and results were similar. Analyses for all loci are presented.

In addition, we obtained SNP data across all seven populations from a subset of 54 individuals using Genotyping-by-Sequencing (GBS)^52^. The 54 individuals are also a subset of 95 individuals from a larger Caribbean wide study. GBS was performed at the Cornell Institute of Genomic Diversity. GBS is a well-standardized approach to collect massive amounts of loci. In brief, DNA samples are barcoded and adapter pairs ligated. Restriction enzymes are then used to fractionate the genomic DNA. An optimization procedure determined that the *PstI* (recognition site: CTGCAG) was the best for the yellowhead jawfish. Once fragments are generated, adapters are ligated to the end of the fragments. Samples are then pooled and size selected to exclude unreacted adapters. Libraries were then amplified using an 18-cycle PCR with long primers complementary to the barcode and common adapters^52^. Samples were run on a 100-bp single-end Illumina HiSeq 2000 lane.

Raw reads were processed using UNEAK, an addition to TASSEL 3.0^53^. UNEAK retains all reads with a barcode, cut site, and no missing data in the first 64 bp after the barcode. Reads are then grouped into tags by 100% identity, tags are aligned pairwise, and any tag pairs differing by one bp are identified as potential SNPs. We identified 1,528,449 total tags after merging, with 639,274 total tag networks identified. To remove sequencing errors, and individuals and SNPs with a high amount of missing data, any alleles represented by a frequency of less than 3% were filtered out.

To further remove data artifacts, we filtered out SNPs that deviated from Hardy–Weinberg equilibrium (p > 0.01) in at least five populations. We initially had over 58,756 SNPs; after removing individuals with <10% of the markers and SNPs with <5% frequency we retained 18,616 SNPs. We also excluded SNPs suspected under selection as identified by BayesScan 2.01^54^ (12 loci) and consider only SNP per tag marker. To overcome any bias associated with having missing SNPs, we first generated a file with no missing information. This file has 90 individuals genotyped at 525 SNPs. In the second dataset we have individuals that have at least 90% of the SNPs and SNPs present in at least 70% of the samples. This last dataset has 54 individuals and 10,047 SNPs.

### Genetic Diversity, Isolation by Distance and Effective Dispersal

We quantified genetic diversity across markers in GENODIVE 2.0^55^ and estimated heterozygosity and number of alleles per population across all 12 microsatellites and 10,047 SNPs. We also estimated genetic variation across geography by calculating pairwise *F*_*ST*_s among locations. We then estimated pairwise linear distance between locations in Google Earth 6.2 using the shortest nautical distance among populations. We then plotted genetic and geographical distances and estimated isolation-by-distance (IBD), assuming a linear regression in R^56^.

An added feature of SNP data is that one can sample thousands of semi-independent markers and estimate more precisely the average genome-wide genetic distance between individuals. This improved power allows estimating IBD patterns using single individuals per population. To test whether single individuals would give similar results than patterns derived from populations, we randomly selected an individual from each population and iterated the process twenty times. We used the ê distance^57^ to measure differences between pairs of individuals.

To estimate effective dispersal, we used Rosset’s (1997) approach:

1 $$\sigma =\sqrt{\frac{1}{4{D}_{e}m}}$$

where *D*_*e*_ is the effective density and *m* is the slope derived from our analysis of IBD. To estimate *D*_*e*_ we used both a direct density estimate of fish per kilometer and one derived from genetic data by estimating effective population size.

Field densities were compiled from studies in Panama^27^, and two locations in Puerto Rico: Rincón and La Parguera^26^. We estimated differences in habitat areas by measuring habitat patch sizes from GIS maps^58^. These estimates are likely biased upwardly and represent the upper bound of the highest fish density.

To measure effective dispersal from genetic data, we used effective population size as implemented in IMa2 (*Θ* = 4 *N*_*e*_ *μ*; where *Θ* is genetic variation in the population; *N*_*e*_ is the effective population size and *μ* is the mutation rate). We ran IMa2 (version 8/27/12)^28^ on 12 microsatellites using as populations La Caleta and Puerto Rico (all populations except La Caleta and PNNE). We run IMa2 for 3,000,000 steps after an initial burnin of 1,000,000 steps. We repeated the process three times and check for convergence on similar results. All ESS values for all runs were above 50. We estimated effective population size as the mean across runs. We used a mutation rate of 5 × 10^−4^ ^29–31^ and one year generation time to estimate population sizes in demographic units. Once we had an estimate of the effective population size, we obtained fish density per km^2^ by dividing by the total coral reef area across the Caribbean (26,000 km^2^)^59^. Our density from genetic data is biased towards the lowest possible density, as long-term effective sizes are lower compared to census size (e.g., for instance in humans *N*_*e*_ is only 10,000 and census is >7 billion;^60^. In addition, we divided *N*_*e*_ by the reef area across the Caribbean, even though the yellowhead jawfish is a habitat specialist and occurs only on colonized pavement with sand channels on coral reefs, which in Puerto Rico make <30% of the reef area^26^. By having the direct census size as the upper bound of the highest density and the genetic inference with the largest possible distributional area, as the lowest possible density, we provide the widest range of effective dispersal estimates.

### Genetic Differentiation and Contemporary Connectivity

To test for genetic differentiation, we used linkage disequilibrium among markers via Bayesian clustering^45^. We ran STRUCTURE 2.3.2 on microsatellites and SNPs assuming an admixture model with a burn-in of 900,000 steps followed by 10 million iterations and 3 replicates per run. We assumed a number of inferred populations from 1 to 7 and then used the Evanno method^61^ to infer the optimal number of populations (K’s) in our dataset. In addition, we run Admixture 1.3^46^ with cross-validation, and 1000 bootstrapping replicates for K values 1, 2, 3, 4 and 5 (Fig. S4). To further understand the distribution of genetic variation across geography, we calculated principal components in GENODIVE 2.0^55^ and principal coordinates in Genalex 6.5^62, 63^. PCA analysis of both SNPs and microsatellites are shown in the main text (Fig. 2) and principal coordinates in the supplement (Fig. S5).

To test for contemporary gene flow, we used BayesAss 3^64^. To analyze our data in BayesAss, we used all seven populations sampled. For each analysis we ran BayesAss for a burn-in of 9,000,000 steps and inferred parameters using the next 90,000,000–300,000,000 iterations sampled at 10,000 intervals. BayesAss is constrained on using <400 loci so we generated 18 random subsets of our data and run each individually. The program was run using different random seed values. Mixing and convergence were inspected using TRACER 1.6^65^. Given the lack of convergence across runs, we also run the program three times with smaller datasets consisting of only populations from Puerto Rico.

### Historical Connectivity

To quantify historical connectivity across the Mona Passage, we used Treemix 1.12^44^ which can handle SNP data without ascertainment bias. To analyze the data in Treemix, the same population partitions were used as in BayesAss. To infer migration parameters, we used La Caleta, DR (western-most population) as the outgroup, as a larger study across the Caribbean suggested it to be less related to all other populations^25^. To increase our confidence in the Treemix estimates we ran the analysis with 500 bootstrap replicates and drew trees in R.

Both contemporary and historical levels of migration inferred from BayesAss^64^ and Treemix^66^ do come with some limitations and assumptions. Among others, that the data are presumably derived from “neutrally” evolving markers and there is independence (not in significant LD) among the markers. We have excluded loci suspected to be under selection as inferred from Bayescan 2.01^54^ (12 outlier loci) and only consider one SNP per tag marker.

## Electronic supplementary material

Supplementary Information
