# Supplementary material for: Effective Dispersal of Caribbean Reef Fish is Smaller than Current Spacing Among Marine Protected Areas
Source: Sci Rep. 2017 Jul 5;7:4689. doi: 10.1038/s41598-017-04849-5 (PMC5498645; doi:10.1038/s41598-017-04849-5)
Supplement: Supplementary file 1 — Supplementary Information [file 41598_2017_4849_MOESM1_ESM.docx]

**Supplementary Information**

**Effective Dispersal of Caribbean Reef Fish is Smaller than Current Spacing Among Marine Protected Areas**

Diana Beltrán^1*^

Nikolaos V Schizas^1^

Richard S Appeldoorn^1^

Carlos Prada^2*^

^1^Department of Marine Sciences, University of Puerto Rico, Mayagüez, Call Box 9000, Mayagüez, 00681, Puerto Rico, USA

^2^Smithsonian Tropical Research Institute, Box 2072, Balboa, Panama

*Corresponding author

**Table S1.** Genetic Diversity for 12 microsatellite loci per locus ^1^.

| **Locus** | **Number of alleles** | **Heterozygosity expected (Hs)** | **Heterozygosity observed (Ho)** | **Total**  **Heterozygosity** **(Ht)** | **H-W**  **Equilibrium** |
| --- | --- | --- | --- | --- | --- |
| **785** | 26 | 0.937 | 0.917 | 0.937 | 0.313 |
| **1588** | 23 | 0.805 | 0.938 | 0.805 | 0.004* |
| **1438** | 16 | 0.889 | 0.938 | 0.889 | 0.254 |
| **8462** | 17 | 0.871 | 0.771 | 0.871 | 0.02 |
| **325** | 37 | 0.933 | 0.938 | 0.933 | 0.441 |
| **170** | 26 | 0.946 | 0.979 | 0.946 | 0.385 |
| **516** | 15 | 0.883 | 1 | 0.883 | 0.005 |
| **150** | 18 | 0.91 | 0.979 | 0.91 | 0.052 |
| **246** | 19 | 0.901 | 0.958 | 0.901 | 0.048 |
| **8537** | 29 | 0.923 | 0.854 | 0.923 | 0.069 |
| **1358** | 38 | 0.94 | 0.979 | 0.94 | 0.335 |
| **7983** | 17 | 0.877 | 0.621 | 0.877 | 0.001* |

- Deviation from Hardy – Weinberg equilibrium after Bonferroni correction (P < 0.0045).

**Table S2.** Genetic Diversity for 12 microsatellite loci per population.

| **Population** | **Number of alleles** | **Heterozygosity Within Populations (Hs)** | **Total Heterozygosity (Ht)** | **Number of Samples** | **H-W**  **Equilibrium** |
| --- | --- | --- | --- | --- | --- |
| **Tres Palmas** | 10.727 | 0.893 | 0.893 | 16 | 0.052 |
| **Parguera** | 14.417 | 0.805 | 0.805 | 55 | 0.989 |
| **PNNE** | 14.667 | 0.895 | 0.895 | 29 | 0.020 |
| **Mona** | 16.833 | 0.879 | 0.879 | 57 | 0.042 |
| **La Caleta** | 13.833 | 0.882 | 0.882 | 28 | 0.604 |
| **Desecheo** | 14.5 | 0.879 | 0.879 | 46 | 0.551 |
| **Culebra** | 13.25 | 0.887 | 0.887 | 29 | 0.082 |

* Deviation from Hardy – Weinberg equilibrium after Bonferroni correction (P < 0.0071).

**Table S3.** Genetic Diversity for 54 individuals, per 10,047 SNPs per population.

| **Population** | **Number of alleles** | **Heterozygosity Within Populations (Hs)** | **Total Heterozygosity (Ht)** | **Number of Samples** | **H-W**  **Equilibrium** |
| --- | --- | --- | --- | --- | --- |
| **Culebra** | 1.438 | 0.118 | 0.118 | 8 | 0.181 |
| **Desecheo** | 1.386 | 0.118 | 0.118 | 6 | 0.153 |
| **La Caleta** | 1.409 | 0.116 | 0.116 | 7 | 0.076 |
| **Mona** | 1.539 | 0.124 | 0.124 | 11 | 0.099 |
| **PNNE** | 1.398 | 0.132 | 0.132 | 5 | 0.083 |
| **Parguera** | 1.492 | 0.122 | 0.122 | 9 | 0.047 |
| **Rincon** | 1.449 | 0.121 | 0.121 | 8 | 0.176 |

**Table S4.** Summary of indices of genetic diversity for 54 individuals, per 10,047 SNPs.

| **Statistic** | **Value** | **Std.Dev.** | **c.i.2.5%** | **c.i.97.5%** |
| --- | --- | --- | --- | --- |
| **Number of alleles** | 1.869 | 0.003 | 1.862 | 1.875 |
| **Effective number of alleles** | 1.156 | 0.002 | 1.152 | 1.161 |
| **Heterozygosity Within Populations** | 0.122 | 0.001 | 0.119 | 0.124 |
| **Total Heterozygosity** | 0.132 | 0.001 | 0.129 | 0.135 |
| **Corrected total Heterozygosity** | 0.134 | 0.001 | 0.131 | 0.137 |

**Table S5.** Proportion of recent migrants as inferred with BayesAss. Columns indicate the source of migrants and raws the receiving population. All iterations varied between 90 to 150 million. Bottom part of the table indicates runs done for populations within Puerto Rico.

|  |  |  | Migrants from |  |  |  |  |
| --- | --- | --- | --- | --- | --- | --- | --- |
|  | **La Caleta** | **PNNE** | **Mona** | **Desecheo** | **Rincon** | **La Parguera** | **Culebra** |
|  | Loci: 350; Populations: 7; Random seed=2122; MCMC iterations=90000000; Burn-in=9000000; Sampling interval=10000 | | | | | | |
| **La Caleta** | 0.8570(0.0426) | 0.0240(0.0223) | 0.0240(0.0223) | 0.0237(0.0222) | 0.0239(0.0223) | 0.0238(0.0221) | 0.0236(0.0219) |
| **PNNE** | 0.0279(0.0259) | 0.6945(0.0257) | 0.1660(0.0461) | 0.0279(0.0255) | 0.0277(0.0254) | 0.0278(0.0256) | 0.0282(0.0260) |
| **Mona** | 0.0186(0.0176) | 0.0187(0.0177) | **0.8886(0.0362)** | 0.0183(0.0174) | 0.0186(0.0176) | 0.0185(0.0176) | 0.0187(0.0178) |
| **Desecheo** | 0.0257(0.0237) | 0.0258(0.0239) | 0.1793(0.0446) | 0.6923(0.0236) | 0.0256(0.0237) | 0.0256(0.0237) | 0.0257(0.0237) |
| **Rincon** | 0.0210(0.0198) | 0.0208(0.0195) | 0.2084(0.0388) | 0.0207(0.0193) | 0.6875(0.0196) | 0.0208(0.0195) | 0.0209(0.0196) |
| **Parguera** | 0.0222(0.0207) | 0.0222(0.0206) | 0.2002(0.0406) | 0.0221(0.0207) | 0.0224(0.0208) | 0.6888(0.0207) | 0.0222(0.0209) |
| **Culebra** | 0.0225(0.0210) | 0.0222(0.0207) | 0.1992(0.0404) | 0.0224(0.0208) | 0.0223(0.0207) | 0.0224(0.0209) | 0.6890(0.0208) |
|  | Loci: 350; Populations: 7; Random seed=22; MCMC iterations=90000000; Burn-in=9000000; Sampling interval=10000 | | | | | | |
| **La Caleta** | 0.8568(0.0427) | 0.0238(0.0220) | 0.0237(0.0222) | 0.0237(0.0220) | 0.0240(0.0224) | 0.0243(0.0225) | 0.0237(0.0221) |
| **PNNE** | 0.0277(0.0256) | 0.6945(0.0256) | 0.0279(0.0258) | 0.0280(0.0258) | 0.0278(0.0256) | 0.1666(0.0466) | 0.0276(0.0255) |
| **Mona** | 0.0185(0.0175) | 0.0184(0.0174) | 0.0184(0.0175) | 0.0183(0.0171) | 0.0186(0.0175) | 0.8891(0.0359) | 0.0186(0.0177) |
| **Desecheo** | 0.0253(0.0236) | 0.0256(0.0236) | 0.0257(0.0239) | 0.6922(0.0236) | 0.0256(0.0238) | 0.1802(0.0447) | 0.0254(0.0236) |
| **Rincon** | 0.0209(0.0197) | 0.0211(0.0198) | 0.0210(0.0196) | 0.0208(0.0196) | 0.6875(0.0196) | 0.2079(0.0391) | 0.0208(0.0195) |
| **Parguera** | 0.0221(0.0207) | 0.0221(0.0205) | 0.6889(0.0209) | 0.0223(0.0211) | 0.0222(0.0208) | 0.2000(0.0408) | 0.0223(0.0207) |
| **Culebra** | 0.0223(0.0209) | 0.0222(0.0208) | 0.0223(0.0210) | 0.0223(0.0209) | 0.0224(0.0210) | 0.1996(0.0413) | 0.6890(0.0209) |
|  | Loci: 350; Populations: 7; Random seed=2922; MCMC iterations=18000000; Burn-in=9000000; Sampling interval=10000 | | | | | | |
| **La Caleta** | 0.8570(0.0426) | 0.0240(0.0223) | 0.0240(0.0223) | 0.0237(0.0222) | 0.0239(0.0223) | 0.0238(0.0221) | 0.0236(0.0219) |
| **PNNE** | 0.0279(0.0259) | 0.6945(0.0257) | 0.1660(0.0461) | 0.0279(0.0255) | 0.0277(0.0254) | 0.0278(0.0256) | 0.0282(0.0260) |
| **Mona** | 0.0186(0.0176) | 0.0187(0.0177) | 0.8886(0.0362) | 0.0183(0.0174) | 0.0186(0.0176) | 0.0185(0.0176) | 0.0187(0.0178) |
| **Desecheo** | 0.0257(0.0237) | 0.0258(0.0239) | 0.1793(0.0446) | 0.6923(0.0236) | 0.0256(0.0237) | 0.0256(0.0237) | 0.0257(0.0237) |
| **Rincon** | 0.0210(0.0198) | 0.0208(0.0195) | 0.2084(0.0388) | 0.0207(0.0193) | 0.6875(0.0196) | 0.0208(0.0195) | 0.0209(0.0196) |
| **Parguera** | 0.0222(0.0207) | 0.0222(0.0206) | 0.2002(0.0406) | 0.0221(0.0207) | 0.0224(0.0208) | 0.6888(0.0207) | 0.0222(0.0209) |
| **Culebra** | 0.0225(0.0210) | 0.0222(0.0207) | 0.1992(0.0404) | 0.0224(0.0208) | 0.0223(0.0207) | 0.0224(0.0209) | 0.6890(0.0208) |
|  | Loci: 375; Populations: 7; Random seed=102; MCMC iterations=90000000; Burn-in=9000000; Sampling interval=10000 | | | | | | |
| **La Caleta** | 0.8569(0.0427) | 0.0240(0.0224) | 0.0239(0.0222) | 0.0238(0.0222) | 0.0239(0.0224) | 0.0238(0.0220) | 0.0237(0.0219) |
| **PNNE** | 0.0278(0.0258) | 0.6943(0.0254) | 0.1668(0.0467) | 0.0278(0.0256) | 0.0278(0.0256) | 0.0275(0.0254) | 0.0281(0.0260) |
| **Mona** | 0.0186(0.0175) | 0.0186(0.0176) | 0.8887(0.0361) | 0.0184(0.0175) | 0.0185(0.0176) | 0.0185(0.0176) | 0.0187(0.0178) |
| **Desecheo** | 0.0258(0.0238) | 0.0260(0.0240) | 0.1786(0.0448) | 0.6924(0.0238) | 0.0258(0.0239) | 0.0259(0.0238) | 0.0255(0.0236) |
| **Rincon** | 0.0210(0.0197) | 0.0208(0.0196) | 0.2081(0.0387) | 0.0208(0.0194) | 0.6876(0.0196) | 0.0207(0.0195) | 0.0210(0.0197) |
| **Parguera** | 0.0222(0.0208) | 0.0221(0.0207) | 0.2001(0.0407) | 0.0222(0.0207) | 0.0223(0.0209) | 0.6889(0.0207) | 0.0222(0.0209) |
| **Culebra** | 0.0225(0.0209) | 0.0223(0.0209) | 0.1992(0.0408) | 0.0224(0.0211) | 0.0223(0.0209) | 0.0224(0.0210) | 0.6889(0.0208) |
|  | Loci: 400; Populations: 7; Random seed=2; MCMC iterations=12000000; Burn-in=9000000; Sampling interval=10000 | | | | | | |
| **La Caleta** | 0.8610(0.0427) | 0.0231(0.0216) | 0.0232(0.0216) | 0.0232(0.0215) | 0.0232(0.0215) | 0.0232(0.0218) | 0.0232(0.0217) |
| **PNNE** | 0.0279(0.0255) | 0.6959(0.0267) | 0.0279(0.0254) | 0.0280(0.0257) | 0.1647(0.0457) | 0.0277(0.0255) | 0.0278(0.0256) |
| **Mona** | 0.0187(0.0176) | 0.0188(0.0176) | 0.6861(0.0182) | 0.0188(0.0176) | 0.2201(0.0365) | 0.0188(0.0178) | 0.0187(0.0177) |
| **Desecheo** | 0.0259(0.0238) | 0.0258(0.0238) | 0.0258(0.0238) | 0.6938(0.0249) | 0.1773(0.0439) | 0.0256(0.0235) | 0.0259(0.0239) |
| **Rincon** | 0.0202(0.0190) | 0.0202(0.0191) | 0.0202(0.0190) | 0.0203(0.0190) | 0.8786(0.0387) | 0.0203(0.0191) | 0.0202(0.0190) |
| **Parguera** | 0.0224(0.0209) | 0.0224(0.0209) | 0.0225(0.0210) | 0.0224(0.0209) | 0.1976(0.0405) | 0.6902(0.0217) | 0.0226(0.0210) |
| **Culebra** | 0.0224(0.0208) | 0.0226(0.0211) | 0.0226(0.0211) | 0.0225(0.0209) | 0.1975(0.0406) | 0.0225(0.0210) | 0.6900(0.0216) |
|  | Loci: 350; Populations: 7; Random seed=912; MCMC iterations=18000000; Burn-in=9000000; Sampling interval=10000 | | | | | | |
| **La Caleta** | 0.8558(0.0430) | 0.0239(0.0222) | 0.0238(0.0222) | 0.0239(0.0222) | 0.0238(0.0222) | 0.0250(0.0232) | 0.0238(0.0222) |
| **PNNE** | 0.0824(0.0459) | 0.6945(0.0256) | 0.0278(0.0255) | 0.0278(0.0256) | 0.0277(0.0254) | 0.1120(0.0492) | 0.0277(0.0254) |
| **Mona** | 0.0185(0.0175) | 0.0185(0.0175) | 0.6852(0.0175) | 0.0185(0.0175) | 0.0186(0.0175) | 0.2222(0.0360) | 0.0185(0.0175) |
| **Desecheo** | 0.0256(0.0237) | 0.0256(0.0238) | 0.0257(0.0238) | 0.6923(0.0237) | 0.0257(0.0237) | 0.1795(0.0444) | 0.0257(0.0238) |
| **Rincon** | 0.0223(0.0207) | 0.0221(0.0207) | 0.0222(0.0208) | 0.0223(0.0209) | 0.6889(0.0208) | 0.2001(0.0409) | 0.0221(0.0207) |
| **Parguera** | 0.0210(0.0198) | 0.0208(0.0196) | 0.0209(0.0196) | 0.0209(0.0196) | 0.0208(0.0196) | 0.8748(0.0392) | 0.0209(0.0196) |
| **Culebra** | 0.0223(0.0209) | 0.0222(0.0207) | 0.0222(0.0208) | 0.0221(0.0207) | 0.0222(0.0207) | 0.2001(0.0408) | 0.6889(0.0208) |
|  | Loci: 100; Populations: 7; Random seed=5622; MCMC iterations=90000000; Burn-in=9000000; Sampling interval=10000 | | | | | | |
| **La Caleta** | 0.8558(0.0430) | 0.0238(0.0222) | 0.0238(0.0222) | 0.0239(0.0222) | 0.0238(0.0222) | 0.0251(0.0234) | 0.0238(0.0221) |
| **PNNE** | 0.0818(0.0460) | 0.6944(0.0255) | 0.0277(0.0255) | 0.0278(0.0255) | 0.0278(0.0255) | 0.1128(0.0494) | 0.0277(0.0255) |
| **Mona** | 0.0185(0.0175) | 0.0185(0.0175) | 0.6851(0.0175) | 0.0185(0.0174) | 0.0185(0.0175) | 0.2225(0.0360) | 0.0184(0.0174) |
| **Desecheo** | 0.0257(0.0238) | 0.0256(0.0237) | 0.0257(0.0238) | 0.6923(0.0237) | 0.0256(0.0237) | 0.1795(0.0444) | 0.0256(0.0237) |
| **Rincon** | 0.0223(0.0208) | 0.0222(0.0207) | 0.0223(0.0208) | 0.0223(0.0209) | 0.6889(0.0209) | 0.1998(0.0408) | 0.0222(0.0207) |
| **Parguera** | 0.0210(0.0197) | 0.0209(0.0196) | 0.0208(0.0195) | 0.0209(0.0196) | 0.0209(0.0197) | 0.8748(0.0391) | 0.0208(0.0195) |
| **Culebra** | 0.0223(0.0207) | 0.0222(0.0209) | 0.0222(0.0208) | 0.0223(0.0209) | 0.0223(0.0208) | 0.1998(0.0408) | 0.6890(0.0208) |
|  | Loci: 381; Populations: 7; Random seed=78; MCMC iterations=20000000; Burn-in=9000000; Sampling interval=10000 | | | | | | |
| **La Caleta** | 0.8481(0.0444) | 0.0241(0.0225) | 0.0335(0.0282) | 0.0234(0.0217) | 0.0234(0.0219) | 0.0241(0.0227) | 0.0235(0.0219) |
| **PNNE** | 0.0278(0.0258) | 0.6944(0.0257) | 0.1665(0.0465) | 0.0278(0.0255) | 0.0279(0.0256) | 0.0277(0.0255) | 0.0279(0.0254) |
| **Mona** | 0.0185(0.0177) | 0.0184(0.0174) | 0.8890(0.0360) | 0.0186(0.0175) | 0.0183(0.0171) | 0.0186(0.0172) | 0.0185(0.0177) |
| **Desecheo** | 0.0257(0.0236) | 0.0251(0.0234) | 0.1789(0.0444) | 0.6921(0.0234) | 0.0259(0.0241) | 0.0260(0.0241) | 0.0264(0.0242) |
| **Rincon** | 0.0224(0.0207) | 0.0223(0.0209) | 0.1998(0.0408) | 0.0220(0.0206) | 0.0222(0.0206) | 0.6893(0.0210) | 0.0221(0.0205) |
| **Parguera** | 0.0206(0.0194) | 0.0207(0.0194) | 0.2085(0.0391) | 0.0210(0.0194) | 0.6874(0.0193) | 0.0211(0.0200) | 0.0206(0.0195) |
| **Culebra** | 0.0221(0.0208) | 0.0221(0.0209) | 0.2000(0.0412) | 0.0221(0.0209) | 0.0223(0.0208) | 0.0222(0.0207) | 0.6892(0.0212) |
|  | Loci: 392; Populations: 7; Random seed=542; MCMC iterations=200000000; Burn-in=9000000; Sampling interval=10000 | | | | | | |
| **La Caleta** | 0.8480(0.0446) | 0.0237(0.0222) | 0.0327(0.0278) | 0.0238(0.0221) | 0.0238(0.0220) | 0.0241(0.0222) | 0.0238(0.0222) |
| **PNNE** | 0.0276(0.0253) | 0.6943(0.0254) | 0.1662(0.0458) | 0.0276(0.0253) | 0.0278(0.0255) | 0.0283(0.0261) | 0.0282(0.0261) |
| **Mona** | 0.0185(0.0174) | 0.0185(0.0176) | 0.8889(0.0361) | 0.0184(0.0173) | 0.0187(0.0176) | 0.0184(0.0175) | 0.0187(0.0177) |
| **Desecheo** | 0.0253(0.0232) | 0.0253(0.0232) | 0.1799(0.0445) | 0.6922(0.0241) | 0.0255(0.0237) | 0.0256(0.0238) | 0.0262(0.0239) |
| **Rincon** | 0.0225(0.0208) | 0.0225(0.0210) | 0.2000(0.0409) | 0.0221(0.0210) | 0.0218(0.0207) | 0.6888(0.0207) | 0.0223(0.0207) |
| **Parguera** | 0.0206(0.0193) | 0.0209(0.0195) | 0.2089(0.0392) | 0.0207(0.0195) | 0.6872(0.0191) | 0.0209(0.0194) | 0.0208(0.0195) |
| **Culebra** | 0.0222(0.0207) | 0.0224(0.0210) | 0.1994(0.0407) | 0.0226(0.0210) | 0.0222(0.0208) | 0.0222(0.0207) | 0.6891(0.0209) |
|  | Loci: 400; Populations: 7; Random seed=3922; MCMC iterations=90000000; Burn-in=9000000; Sampling interval=10000 | | | | | | |
| **La Caleta** | 0.8559(0.0429) | 0.0239(0.0222) | 0.0238(0.0221) | 0.0239(0.0222) | 0.0249(0.0233) | 0.0240(0.0223) | 0.0237(0.0220) |
| **PNNE** | 0.0823(0.0457) | 0.6945(0.0255) | 0.0279(0.0255) | 0.0278(0.0255) | 0.1120(0.0492) | 0.0278(0.0256) | 0.0277(0.0255) |
| **Mona** | 0.0184(0.0175) | 0.0185(0.0175) | 0.6852(0.0176) | 0.0185(0.0174) | 0.2223(0.0361) | 0.0185(0.0176) | 0.0185(0.0175) |
| **Desecheo** | 0.0256(0.0237) | 0.0257(0.0237) | 0.0256(0.0237) | 0.6925(0.0239) | 0.1795(0.0445) | 0.0255(0.0237) | 0.0256(0.0237) |
| **Rincon** | 0.0210(0.0199) | 0.0209(0.0197) | 0.0209(0.0196) | 0.0209(0.0196) | 0.8747(0.0392) | 0.0209(0.0197) | 0.0207(0.0195) |
| **Parguera** | 0.0221(0.0207) | 0.0222(0.0207) | 0.0221(0.0207) | 0.0222(0.0206) | 0.2003(0.0409) | 0.6889(0.0208) | 0.0222(0.0208) |
| **Culebra** | 0.0222(0.0206) | 0.0223(0.0208) | 0.0221(0.0207) | 0.0223(0.0208) | 0.1999(0.0407) | 0.0222(0.0207) | 0.6890(0.0209) |
|  | Loci: 100; Populations: 7; Random seed=8522; MCMC iterations=200000000; Burn-in=9000000; Sampling interval=10000 | | | | | | |
| **La Caleta** | 0.8560(0.0430) | 0.0237(0.0221) | 0.0237(0.0221) | 0.0239(0.0222) | 0.0250(0.0232) | 0.0239(0.0222) | 0.0237(0.0220) |
| **PNNE** | 0.0835(0.0459) | 0.6945(0.0256) | 0.0279(0.0257) | 0.0276(0.0255) | 0.1111(0.0489) | 0.0277(0.0255) | 0.0278(0.0256) |
| **Mona** | 0.0186(0.0176) | 0.0185(0.0174) | 0.6853(0.0176) | 0.0186(0.0175) | 0.2217(0.0357) | 0.0187(0.0176) | 0.0186(0.0176) |
| **Desecheo** | 0.0255(0.0236) | 0.0256(0.0238) | 0.0258(0.0239) | 0.6922(0.0236) | 0.1796(0.0445) | 0.0257(0.0238) | 0.0256(0.0237) |
| **Rincon** | 0.0209(0.0197) | 0.0207(0.0195) | 0.0209(0.0196) | 0.0208(0.0195) | 0.8749(0.0392) | 0.0209(0.0196) | 0.0209(0.0196) |
| **Parguera** | 0.0223(0.0208) | 0.0222(0.0207) | 0.0222(0.0209) | 0.0222(0.0208) | 0.2000(0.0408) | 0.6889(0.0207) | 0.0222(0.0208) |
| **Culebra** | 0.0221(0.0208) | 0.0223(0.0209) | 0.0223(0.0208) | 0.0223(0.0207) | 0.1999(0.0407) | 0.0223(0.0209) | 0.6889(0.0207) |
|  | Loci: 380; Populations: 7; Random seed=7322; MCMC iterations=90000000; Burn-in=9000000; Sampling interval=10000 | | | | | | |
| **La Caleta** | 0.8453(0.0443) | 0.0234(0.0216) | 0.0362(0.0293) | 0.0238(0.0221) | 0.0236(0.0219) | 0.0239(0.0223) | 0.0238(0.0222) |
| **PNNE** | 0.0273(0.0250) | 0.6943(0.0254) | 0.1671(0.0455) | 0.0280(0.0254) | 0.0278(0.0256) | 0.0279(0.0255) | 0.0276(0.0252) |
| **Mona** | 0.0185(0.0174) | 0.0186(0.0175) | 0.8886(0.0362) | 0.0187(0.0178) | 0.0187(0.0177) | 0.0187(0.0178) | 0.0184(0.0175) |
| **Desecheo** | 0.0259(0.0240) | 0.0258(0.0239) | 0.1789(0.0445) | 0.6925(0.0239) | 0.0258(0.0240) | 0.0255(0.0235) | 0.0255(0.0236) |
| **Rincon** | 0.0223(0.0208) | 0.0223(0.0209) | 0.1998(0.0410) | 0.0223(0.0209) | 0.6889(0.0209) | 0.0223(0.0210) | 0.0222(0.0207) |
| **Parguera** | 0.0211(0.0198) | 0.0209(0.0196) | 0.2084(0.0389) | 0.0208(0.0196) | 0.0205(0.0191) | 0.6875(0.0196) | 0.0207(0.0195) |
| **Culebra** | 0.0221(0.0207) | 0.0223(0.0208) | 0.2001(0.0407) | 0.0223(0.0209) | 0.0223(0.0209) | 0.0221(0.0205) | 0.6888(0.0207) |
|  | Loci: 100; Populations: 7; Random seed=82; MCMC iterations=90000000; Burn-in=9000000; Sampling interval=10000 | | | | | | |
| **La Caleta** | 0.8560(0.0427) | 0.0238(0.0222) | 0.0238(0.0222) | 0.0238(0.0221) | 0.0249(0.0230) | 0.0239(0.0222) | 0.0239(0.0223) |
| **PNNE** | 0.0827(0.0461) | 0.6945(0.0257) | 0.0278(0.0256) | 0.0277(0.0255) | 0.1116(0.0492) | 0.0278(0.0255) | 0.0279(0.0255) |
| **Mona** | 0.0184(0.0174) | 0.0184(0.0174) | 0.6852(0.0176) | 0.0184(0.0174) | 0.2227(0.0362) | 0.0184(0.0175) | 0.0184(0.0175) |
| **Desecheo** | 0.0258(0.0239) | 0.0257(0.0237) | 0.0257(0.0239) | 0.6924(0.0238) | 0.1791(0.0444) | 0.0255(0.0236) | 0.0257(0.0239) |
| **Rincon** | 0.0209(0.0196) | 0.0208(0.0196) | 0.0208(0.0196) | 0.0209(0.0197) | 0.8750(0.0391) | 0.0209(0.0196) | 0.0207(0.0195) |
| **Parguera** | 0.0223(0.0208) | 0.0222(0.0207) | 0.0223(0.0207) | 0.0223(0.0209) | 0.2000(0.0408) | 0.6888(0.0208) | 0.0222(0.0207) |
| **Culebra** | 0.0224(0.0209) | 0.0222(0.0208) | 0.0222(0.0208) | 0.0223(0.0210) | 0.1995(0.0408) | 0.0222(0.0208) | 0.6890(0.0209) |
|  | Loci: 350; Populations: 7; Random seed=412; MCMC iterations=120000000; Burn-in=9000000; Sampling interval=10000 | | | | | | |
| **La Caleta** | 0.8558(0.0430) | 0.0240(0.0222) | 0.0251(0.0233) | 0.0240(0.0223) | 0.0237(0.0221) | 0.0237(0.0222) | 0.0237(0.0221) |
| **PNNE** | 0.0828(0.0461) | 0.6944(0.0256) | 0.1115(0.0494) | 0.0278(0.0255) | 0.0278(0.0255) | 0.0278(0.0255) | 0.0280(0.0259) |
| **Mona** | 0.0190(0.0181) | 0.0184(0.0175) | 0.8888(0.0362) | 0.0184(0.0174) | 0.0184(0.0175) | 0.0185(0.0175) | 0.0185(0.0175) |
| **Desecheo** | 0.0256(0.0237) | 0.0257(0.0239) | 0.1797(0.0444) | 0.6922(0.0236) | 0.0255(0.0237) | 0.0256(0.0237) | 0.0256(0.0238) |
| **Rincon** | 0.0208(0.0195) | 0.0208(0.0196) | 0.2084(0.0391) | 0.0209(0.0195) | 0.6875(0.0196) | 0.0208(0.0196) | 0.0208(0.0195) |
| **Parguera** | 0.0222(0.0207) | 0.0224(0.0209) | 0.1998(0.0410) | 0.0221(0.0208) | 0.0223(0.0209) | 0.6889(0.0207) | 0.0223(0.0209) |
| **Culebra** | 0.0220(0.0206) | 0.0223(0.0208) | 0.2002(0.0408) | 0.0221(0.0206) | 0.0223(0.0209) | 0.0222(0.0209) | 0.6889(0.0209) |
|  | Loci: 400; Populations: 7; Random seed=56822; MCMC iterations=150000000; Burn-in=9000000; Sampling interval=10000 | | | | | | |
| **La Caleta** | 0.6902(0.0218) | 0.0225(0.0210) | 0.0225(0.0211) | 0.1974(0.0409) | 0.0225(0.0210) | 0.0224(0.0209) | 0.0225(0.0210) |
| **PNNE** | 0.0258(0.0237) | 0.6938(0.0247) | 0.0257(0.0237) | 0.1772(0.0437) | 0.0258(0.0238) | 0.0259(0.0237) | 0.0258(0.0237) |
| **Mona** | 0.0239(0.0222) | 0.0241(0.0223) | 0.6918(0.0231) | 0.1881(0.0423) | 0.0240(0.0223) | 0.0240(0.0222) | 0.0241(0.0224) |
| **Desecheo** | 0.0179(0.0170) | 0.0180(0.0170) | 0.0180(0.0171) | 0.8922(0.0353) | 0.0181(0.0171) | 0.0180(0.0172) | 0.0178(0.0170) |
| **Rincon** | 0.0277(0.0254) | 0.0279(0.0255) | 0.0279(0.0256) | 0.1644(0.0458) | 0.6962(0.0268) | 0.0279(0.0257) | 0.0280(0.0257) |
| **Parguera** | 0.0210(0.0197) | 0.0211(0.0199) | 0.0210(0.0196) | 0.2060(0.0391) | 0.0211(0.0197) | 0.6887(0.0204) | 0.0211(0.0197) |
| **Culebra** | 0.0226(0.0210) | 0.0224(0.0208) | 0.0224(0.0209) | 0.1979(0.0407) | 0.0224(0.0209) | 0.0224(0.0208) | 0.6900(0.0216) |
|  | Loci: 150; Populations: 7; Random seed=7622; MCMC iterations=90000000; Burn-in=9000000; Sampling interval=10000 | | | | | | |
| **La Caleta** | 0.8704(0.0407) | 0.0216(0.0202) | 0.0216(0.0202) | 0.0217(0.0204) | 0.0215(0.0202) | 0.0216(0.0203) | 0.0217(0.0203) |
| **PNNE** | 0.1772(0.0439) | 0.6936(0.0247) | 0.0260(0.0239) | 0.0258(0.0239) | 0.0257(0.0237) | 0.0259(0.0238) | 0.0257(0.0237) |
| **Mona** | 0.0246(0.0229) | 0.0232(0.0216) | 0.8596(0.0430) | 0.0232(0.0217) | 0.0232(0.0217) | 0.0231(0.0216) | 0.0231(0.0216) |
| **Desecheo** | 0.2200(0.0366) | 0.0188(0.0178) | 0.0187(0.0177) | 0.6861(0.0184) | 0.0189(0.0178) | 0.0188(0.0178) | 0.0187(0.0177) |
| **Rincon** | 0.1637(0.0461) | 0.0278(0.0256) | 0.0291(0.0266) | 0.0279(0.0257) | 0.6960(0.0267) | 0.0278(0.0254) | 0.0278(0.0256) |
| **Parguera** | 0.2060(0.0394) | 0.0210(0.0198) | 0.0212(0.0199) | 0.0211(0.0196) | 0.0211(0.0198) | 0.6886(0.0204) | 0.0211(0.0198) |
| **Culebra** | 0.1975(0.0405) | 0.0226(0.0210) | 0.0225(0.0209) | 0.0226(0.0210) | 0.0224(0.0208) | 0.0224(0.0209) | 0.6901(0.0217) |
|  | Loci: 300; Populations: 7; Random seed=898; MCMC iterations=300000000; Burn-in=9000000; Sampling interval=10000 | | | | | | |
| **La Caleta** | 0.6901(0.0219) | 0.0225(0.0210) | 0.0225(0.0210) | 0.0225(0.0211) | 0.0225(0.0209) | 0.1973(0.0407) | 0.0227(0.0211) |
| **PNNE** | 0.0256(0.0237) | 0.6938(0.0248) | 0.0259(0.0240) | 0.0259(0.0240) | 0.0257(0.0236) | 0.1773(0.0438) | 0.0259(0.0239) |
| **Mona** | 0.0231(0.0214) | 0.0231(0.0216) | 0.8610(0.0426) | 0.0233(0.0217) | 0.0232(0.0216) | 0.0232(0.0217) | 0.0231(0.0214) |
| **Desecheo** | 0.0186(0.0177) | 0.0187(0.0176) | 0.0187(0.0177) | 0.6860(0.0183) | 0.0187(0.0177) | 0.2204(0.0368) | 0.0188(0.0177) |
| **Rincon** | 0.0277(0.0254) | 0.0277(0.0255) | 0.0280(0.0258) | 0.0277(0.0254) | 0.6962(0.0268) | 0.1647(0.0458) | 0.0279(0.0257) |
| **Parguera** | 0.0201(0.0189) | 0.0201(0.0190) | 0.0201(0.0189) | 0.0203(0.0190) | 0.0202(0.0191) | 0.8788(0.0389) | 0.0204(0.0192) |
| **Culebra** | 0.0224(0.0208) | 0.0224(0.0209) | 0.0226(0.0210) | 0.0223(0.0207) | 0.0224(0.0209) | 0.1977(0.0404) | 0.6900(0.0217) |
|  | Loci: 400; Populations: 7; Random seed=722; MCMC iterations=200000000; Burn-in=9000000; Sampling interval=10000 | | | | | | |
| **La Caleta** | 0.6901(0.0217) | 0.0225(0.0210) | 0.0225(0.0210) | 0.1974(0.0407) | 0.0226(0.0211) | 0.0224(0.0208) | 0.0225(0.0210) |
| **PNNE** | 0.0256(0.0236) | 0.6936(0.0247) | 0.0259(0.0238) | 0.1776(0.0437) | 0.0258(0.0238) | 0.0258(0.0238) | 0.0256(0.0237) |
| **Mona** | 0.0231(0.0216) | 0.0230(0.0215) | 0.8615(0.0425) | 0.0232(0.0216) | 0.0231(0.0217) | 0.0231(0.0214) | 0.0229(0.0214) |
| **Desecheo** | 0.0179(0.0170) | 0.0180(0.0170) | 0.0179(0.0171) | 0.8923(0.0355) | 0.0180(0.0171) | 0.0179(0.0171) | 0.0179(0.0170) |
| **Rincon** | 0.0278(0.0256) | 0.0278(0.0255) | 0.0279(0.0255) | 0.1647(0.0457) | 0.6959(0.0265) | 0.0278(0.0255) | 0.0280(0.0256) |
| **Parguera** | 0.0211(0.0198) | 0.0211(0.0198) | 0.0212(0.0198) | 0.2059(0.0389) | 0.0210(0.0197) | 0.6886(0.0205) | 0.0211(0.0198) |
| **Culebra** | 0.0224(0.0208) | 0.0224(0.0210) | 0.0225(0.0210) | 0.1980(0.0407) | 0.0224(0.0210) | 0.0223(0.0209) | 0.6900(0.0216) |
|  | Loci: 400; Populations: 5; Random seed=1; MCMC iterations=120000000; Burn-in=9000000; Sampling interval=10000 | | | | | | |
| **Mona** |  |  | 0.6875(0.0197) | 0.0207(0.0194) | 0.2501(0.0349) | 0.0208(0.0195) | 0.0208(0.0195) |
| **Desecheo** |  |  | 0.0304(0.0277) | 0.6969(0.0277) | 0.2123(0.0462) | 0.0303(0.0276) | 0.0301(0.0276) |
| **Rincon** |  |  | 0.0237(0.0222) | 0.0238(0.0222) | 0.9049(0.0388) | 0.0238(0.0222) | 0.0238(0.0222) |
| **Parguera** |  |  | 0.0256(0.0237) | 0.0257(0.0237) | 0.2307(0.0412) | 0.6924(0.0238) | 0.0257(0.0238) |
| **Culebra** |  |  | 0.0255(0.0237) | 0.0255(0.0236) | 0.2310(0.0411) | 0.0258(0.0238) | 0.6923(0.0236) |
|  | Loci: 400; Populations: 5; Random seed=2; MCMC iterations=120000000; Burn-in=9000000; Sampling interval=10000 | | | | | | |
| **Mona** |  |  | 0.6877(0.0197) | 0.0207(0.0195) | 0.0209(0.0196) | 0.2500(0.0350) | 0.0207(0.0195) |
| **Desecheo** |  |  | 0.0304(0.0278) | 0.6969(0.0276) | 0.0303(0.0277) | 0.2122(0.0460) | 0.0302(0.0276) |
| **Rincon** |  |  | 0.0239(0.0222) | 0.0238(0.0221) | 0.0239(0.0222) | 0.9047(0.0389) | 0.0237(0.0221) |
| **Parguera** |  |  | 0.0256(0.0238) | 0.0256(0.0237) | 0.6924(0.0238) | 0.2307(0.0413) | 0.0257(0.0239) |
| **Culebra** |  |  | 0.0256(0.0238) | 0.0256(0.0238) | 0.0257(0.0238) | 0.2308(0.0411) | 0.6923(0.0237) |
|  | Loci: 400; Populations: 5; Random seed=3; MCMC iterations=120000000; Burn-in=9000000; Sampling interval=10000 | | | | | | |
| **Mona** |  |  | 0.2498(0.0351) | 0.0209(0.0196) | 0.6876(0.0197) | 0.0209(0.0196) | 0.0208(0.0195) |
| **Desecheo** |  |  | 0.2120(0.0464) | 0.6971(0.0277) | 0.0303(0.0275) | 0.0302(0.0277) | 0.0304(0.0278) |
| **Rincon** |  |  | 0.9047(0.0388) | 0.0239(0.0221) | 0.0238(0.0221) | 0.0238(0.0222) | 0.0238(0.0222) |
| **Parguera** |  |  | 0.2306(0.0412) | 0.0257(0.0238) | 0.0257(0.0237) | 0.6924(0.0238) | 0.0257(0.0237) |
| **Culebra** |  |  | 0.2307(0.0410) | 0.0257(0.0238) | 0.0255(0.0236) | 0.0256(0.0237) | 0.6925(0.0239) |
|  | Loci: 400; Populations: 5; Random seed=4; MCMC iterations=120000000; Burn-in=9000000; Sampling interval=10000 | | | | | | |
| **Mona** |  |  | 0.6875(0.0195) | 0.0208(0.0196) | 0.2498(0.0351) | 0.0209(0.0196) | 0.0210(0.0198) |
| **Desecheo** |  |  | 0.0304(0.0276) | 0.6970(0.0276) | 0.2122(0.0461) | 0.0302(0.0276) | 0.0302(0.0275) |
| **Rincon** |  |  | 0.0238(0.0222) | 0.0238(0.0221) | 0.9046(0.0389) | 0.0238(0.0221) | 0.0239(0.0222) |
| **Parguera** |  |  | 0.0257(0.0238) | 0.0255(0.0237) | 0.2309(0.0410) | 0.6922(0.0236) | 0.0257(0.0238) |
| **Culebra** |  |  | 0.0255(0.0236) | 0.0256(0.0236) | 0.2309(0.0411) | 0.0256(0.0237) | 0.6924(0.0238) |

**Table S6.** Parameter estimates and boundaries of the 95% highest posterior density (HPD) for the Isolation with Migration model between the Dominican Republic (La Caleta only) and Puerto Rico. The mutation rate per locus used is: 5 x 10^-4^. Generation time was 1 year. The average population size considering both Puerto Rico and the Dominican Republic across all three runs is: **5’119,320** +/- 1’017,191. To run IMa2 we used: IMa2 –I <input> -o <output> -d1000 -q90 -m200 -t2 -hfg -hn50 -ha0.96 -hb0.9 -u1 -b1000000 -l3000 -p2345.

|  | **Effective size (*N_e_*) Dominican Republic (La Caleta)** | **Effective size (*N_e_*) Puerto Rico** | **Effective size (*N_e_*) ancestral population** |
| --- | --- | --- | --- |
|  |  |  |  |
| **High Point** | **7709071** | **9062321** | **1119296** |
| HPD95Lo | 2507515 | 944223 | 217639 |
| HPD95Hi | 30816020 | 34318231 | 3418231 |
|  |  |  |  |
| **High Point** | **6516616** | **8086461** | **1610094** |
| HPD95Lo | 1861128 | 1745382 | 902898 |
| HPD95Hi | 27442899 | 28741405 | 5817943 |
|  |  |  |  |
| **High Point** | **4590967** | **5637300** | **1741755** |
| HPD95Lo | 1000290 | 713325 | 215859 |
| HPD95Hi | 24892137 | 31892413 | 4097719 |

**Figure S1.** Geographic localities across The Mona Passage. Note that the size of the Reserves in Desecheo, Tres Palmas, and Canal Luis Peña Natural Reserves is too small to be perceived in the map. Map was created *de novo* in ArcGIS 10.3 ^2^ (<http://desktop.arcgis.com/en/arcmap/>).

**Figure S2.** Isolation by Distance (IBD) in *Opistognathus aurifrons* from five sampling sites within Puerto Rico (Mona Island, Desecheo Island, Tres Palmas ,La Paguera, and Culebra). **A.** IBD from 12 microsatellites. **B.** IBD from 10,047 SNPs.

**
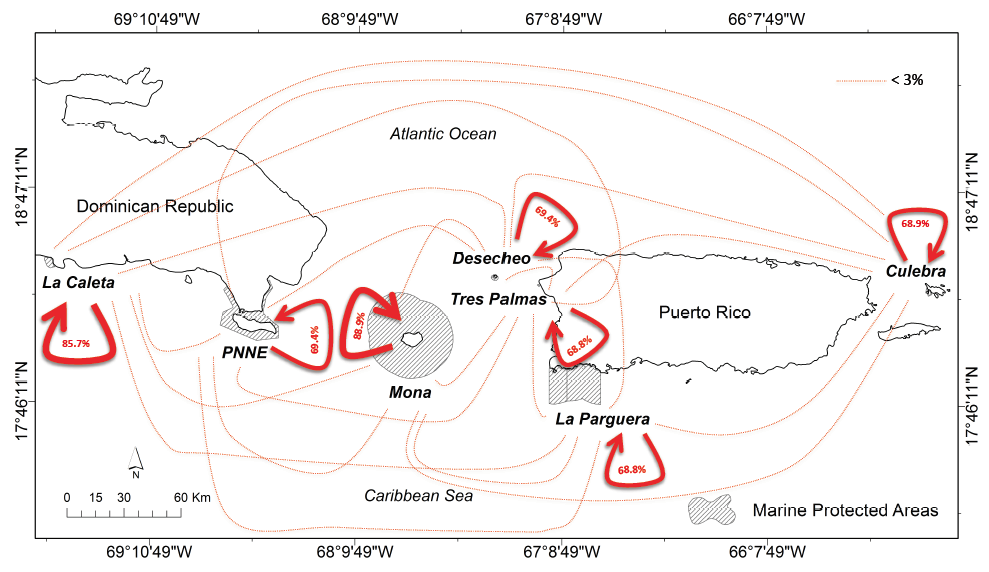
**

**Figure S3.** Contemporary gene flow, and inbreeding patterns analysis results for *Opistognathus aurifrons* among seven MPAs sampling sites populations along the Mona Passage as obtained from BayeAss. Map was created *de novo* in ArcGIS 10.3 ^2^ (<http://desktop.arcgis.com/en/arcmap/>). We caution the reader that runs in BayesAss did not converge into the same solution. While the self-seeding pattern is consistent, the export of migrant from Mona is not always observed across runs (see also Table S5).

**Figure S4.** Admixture results using 10,047 SNPs and 2,000 bootstrap replicates.

**A**

**B**

**12 Microsatellites**

**10,047 SNPs**

**Coordinate 1**

**- 0.4 -0.2 0 -0.2**

**- 0.3 -0.2 - 0.1 0.0 0.1 0.2 0.3**

**Coordinate 2**

**- 0.06 -0.04 - 0.02 0.00 0.02**

**-0.020 - 0.010 0.00**

**La Caleta**

**PNNE**

**Mona**

**Desecheo**

**Tres Palmas**

**La Parguera**

**Culebra**

**Figure S5.** Multidimensional coordinate analysis of genetic variation per individual using 12 microsatellites (A) and 10,047 SNPs (B).

1 Beltrán, D., Schizas, N. V., Prada, C. & Appeldoorn, R. S. Isolation and characterization of 12 microsatellite loci to study connectivity in the yellow jawfish *Opistognathus aurifrons*. *Conservation Genetic Resources* **7**, 543-545, doi:DOI 10.1007/s12686-014-0419-x (2014).

2 ESRI. *ArcGIS Desktop: Release 10*. (Redlands CA: Environmental Systems Research Institute, 2011).
